# Bioaugmentation of biofloc system with enzymatic bacterial strains for high health and production performance of *Penaeus indicus*

**DOI:** 10.1038/s41598-021-93065-3

**Published:** 2021-07-01

**Authors:** A. Panigrahi, P. Esakkiraj, Rashmi Ranjan Das, C. Saranya, T. N. Vinay, S. K. Otta, M. Shashi Shekhar

**Affiliations:** ICAR-Central Institute of Brackishwater Aquaculture, 75, Santhome High Road, Raja Annamalaipuram, Chennai, 600 028 India

**Keywords:** Biotechnology, Immunology, Microbiology

## Abstract

The beneficial effects of two probiotic bacterial strains *Marinilactibacillus piezotolerans* and *Novosphingobium* sp. during the culture of Indian white shrimp, *Penaeus indicus,* under biofloc and clear water system were evaluated. The experimental variation were CW1 (*M. piezotolerans* in clear water), BFT1 (biofloc + *M. piezotolerans*), CW2 (*Novosphingobium* sp. in clear water), BFT2 (biofloc + *Novosphingobium* sp.) and control (without bacterial strains and biofloc). Growth and survival considerably increased in probiotic bio-augmented treatments. Probiotic incorporation significantly improved water quality, especially ammonia reduction. Microbiota analysis from gut samples taken from different treatments revealed varied microbial population structure among clear water culture, biofloc culture and control. *Proteobacteria* and *Firmicutes* were the top phyla observed in the treatments which were significantly higher in bio-augmented systems than the control. *Vibrio* genera were predominantly observed in control and clear water system compared to that of biofloc systems. Immune genes were significantly altered in response to probiotic gut microbial supplementation than the control. Higher gene expression profile of important immune genes was observed in the biofloc reared shrimps. Expression of digestive enzyme related genes such as trypsin, chymotrypsin, cathepsin L, cathepsin B and alpha amylase were also upregulated significantly in probiotic supplementation especially in the biofloc treatments. Proteomic analysis of hepatopancreas of shrimps from different treatments was carried out by using 2D gel electrophoresis and MALDI-TOF analysis. The proteins were mostly related to growth and stress tolerance. Eukaryotic initiation factor 4E binding protein was expressed in all the groups and it was high in biofloc treated animals followed by animals treated solely with probiotics compared to those of control groups. The results concludes that biofloc already proved as an effective culture method for healthy shrimp production and supplementation of probiotic bacterial strains registered additional benefit for growth, survival, microbial, immunological status of *P, indicus* culture.

## Introduction

Aquaculture industry necessitates an eco-friendly and a sustainable approach to increase productivity for the global demand of aquaculture products^1,2^_._ The technique should be advantageous for the environment and as well for the farmer’s^3–5^_. O_ne such technique popularized among the farmer’s in the past two decades is minimal or zero water exchange called biofloc technology. With minimal or zero water exchange, the water quality improves by microbial aggregates developed for the utilization purpose of external supplemented carbon sources. These microbial aggregates facilitate the degradation of organic wastes and assimilation into nutrient material under intense aeration^6–9^. The main advantage of the system is heterogeneous microbial aggregates and it can be developed by addition of carbon sources, because nitrogen concentration from feed and other sources needs to be balanced with addition of carbon sources. Use of low-cost carbon sources and by products such as molasses, rice bran etc. is more helpful to reduce costs and make biofloc technology more viable to the farmers^10^. Also, supplying suitable carbon sources makes it easy to aggregate the heterogeneous microorganisms including bacteria, microalgae, planktons etc. to form a floc with the help of self-produced extracellular polymeric substances.

Biofloc system of aquaculture requires continuous supplying of natural bioactive compounds and other beneficial molecules produced by the microbial aggregates^11^. For this, microbial aggregates imbibe the nitrogenous compounds from feed and other origin as a main source of energy. By this approach, it continuously avoids ammonia and accumulation of other nitrogenous toxins. Also, the consumption of these bioactive molecules has a direct bearing on the physiological and health status of shrimp^12^.

Probiotics are live microbial supplements used in aquaculture to control diseases and also to improve the growth and other related parameters^13,14^. The probiotic supplements are mono or a mixture of beneficial bacterial population having the properties of enzyme production, antimicrobial compound production, and water quality maintenance^13^. Many probiotic strains have been identified, which have a prolonged effect on shrimp culture either by their ability to produce extracellular enzymes such as amylase, protease, lipase. So as to aid the digestive status of shrimp or by producing antimicrobial compounds against invading pathogens^15^. They improve the growth, survival and health status of the host shrimp^16,17^. However, there is less information related to the supplementation of enzymatic probiotic strains into the biofloc system and the combined administration which might yield more appropriate response in shrimp culture. The method of 2D gel electrophoresis combined with MALDI-TOF analysis is one the common efficient techniques which is used to identify the experimental variations at the proteomic level. By using these combinations of methods, many studies identified the differentially expressed proteins in many shrimp species during viral and bacterial infections, temperature stress, dietary changes, hypoxia stress^18–22^, but limited studies are available with respect to these innovative immunomodulatory interventions like biofloc system. To address these issues, the present study was carried out to evaluate growth, immune, gut microbiota and proteomic changes of shrimp (*Penaeus indicus*) cultured in a biofloc based system bio-augmented with two gut isolated enzymatic probiotic strains, compared with shrimp reared in a clear water culture system.

## Results and discussion

**Figure 1:**
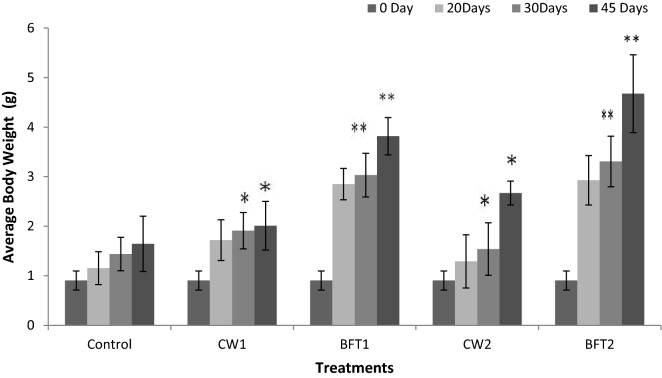
Growth performance of *P. indicus* reared in different probiotic and biofloc systems and error bar showing standard deviation of three replicates. Significance between different groups (*P* < 0.05) marked with asterisk**.**

The effect of bio-augmentation of *Marinilactibacillus piezotolerans* and *Novosphingobium* sp. were studied during the culture of *P. indicus* in biofloc system and the results compared with those from clear water system culture. The results revealed that biofloc culture system is an effective treatment system in terms of growth and survival. These two strains promoted the growth of shrimp significantly (*P* < 0.05) compared to the control (Fig. 1). Growth increased substantially in the BFT-augmented groups (BFT1 and BFT2) and a twofold higher growth was observed in combination with biofloc system. Amongst these two strains, *M. piezotolerans* bio-augmented BFT system was found to be more effective compared to the other treatments. Also, the survival was significantly higher (*P* < 0.05) in strains bio-augmented groups compared to control (82%). The survival was 85, 90, 86 and 91% respectively for CW1, BFT1, CW2 and BFT 2 groups.

By using biofloc technology, intensive shrimp culture can be carried out with reduced water usage, pathogen free and reduced waste output^23,24^. In shrimp, biofloc rearing has been extensively standardized with *P. vannamei* in terms of growth, water quality and health^8,9,13,25–27^. This technology can be popularized further by adopting it to other species of shrimps. Our study revealed that the addition of live microbial supplements considerably improved the performance in terms of growth and survival. Scanty information is available on the use of probiotic with biofloc system. Krummenauer et al.^28^ reported that the supplementation of probiotic mixture in biofloc system significantly improved the growth and survival of *P. vannamei* infected with *V. parahaemolyticus*. A similar trend was observed in an earlier study^14^ conducted by bioaugmenting several probiotic strains in BFT system. The findings in the present study also corroborate the same. Llario et al.^29^ reported that the addition of probiotic strain *B. amyloliquefaciens* directly into biofloc water enhanced the immunity of *P. vannamei* culture; however, there was no effect on growth. In the present study, growth and immunity significantly increased when BFT system was augmented with enzymatic gut microflora as probiotics supplement.

Water quality parameters improved substantially in the biofloc groups compared to the control. Ammonia level was significantly (*P* < 0.05) reduced in strain supplemented BFT groups (BFT1 and 2) than strain supplemented clear water groups (CW1 and 2). Other parameters such as dissolved oxygen level were maintained better in BFT groups than the clear water system and control. Not much variation was observed in other parameters such as pH, salinity among the treatments (Table 1). The carbonate, bicarbonate and hardness levels varied between experimental groups especially in BFT1, CW2 and BFT2 due to restricted water exchange and addition of probiotics and lime. Higher carbonate and bicarbonate in the biofloc treatments indicate a higher alkalinity level is maintained in BFT groups, which gives more buffering capacity compared to control. Higher buffering capacity will always lower diurnal fluctuations of pH and other parameters in the system. However, among the treatments depending on the nature of the probiotics used or presence of different heterotrophic bacterial community, there is non-significant variations in these parameters. Generally biofloc system is known for maintaining the water quality by continuous recycling of nitrogenous wastes generated from the culture system^6^. The strains selected as a supplement were observed to produce different kind hydrolytic enzymes^31^ and this will give additional support for the nitrogen recycling and avoids the toxic deposition in the culture system.

**Table 1:** Water quality parameters observed in different treatments (mean ± SE (n)).

Parameter	Control	CW1	BFT1	CW2	BFT2
Nitrite	1.132 ^b^ ± 0.12	1.097^b^ ± 0.1	0.509^a^ ± 0.07	0.638^a^ ± 0.09	0.505^a^ ± 0.06
Ammonia	0.675^c^ ± 0.09	0.312^b^ ± 0.05	0.014^a^ ± 0.002	0.271^b^ ± 0.01	0.006^a^ ± 0.001
Phosphate	1.549 ^a^ ± 0.1	1.336 ^a^ ± 0.2	1.712^a^ ± 0.15	1.618^a^ ± 0.18	2.087^b^ ± 0.16
Carbonate	0.2^a^ ± 0.01	0.5^a^ ± 0.06	4.7^bc^ ± 0.12	4.3^b^ ± 0.2	5^c^ ± 0.28
Bicarbonate	28.7 ^a^ ± 1.2	32.3^a^ ± 2.5	76.8^b^ ± 3.1	74.4^b^ ± 3.5	79.6^b^ ± 3.2
Hardness	8.4^a^ ± 1.0	14.7^b^ ± 1.8	42^c^ ± 3.4	37^c^ ± 2.9	47.8^d^ ± 3
pH	7.8^a^ ± 0.2	7.5^a^ ± 0.3	7.9^a^ ± 0.1	7.8^a^ ± 0.1	7.6 ^a^ ± 0.2
Dissolved oxygen	5.9^a^ ± 0.1	6.^ab^ ± 0.3	6.6^bc^ ± 0.2	6.2^ab^ ± 0.4	6.7^c^ ± 0.1
Salinity	35^b^ ± 1	34^ab^ ± 1	33^a^ ± 2	34^ab^ ± 2	35^b^ ± 1

Means in the same row having different superscript^a,b,c,d^ differ significantly. *P < 0.05; Means in the same row having same superscript are not significant.

### Analysis of gut microbiota

**Figure 2:**
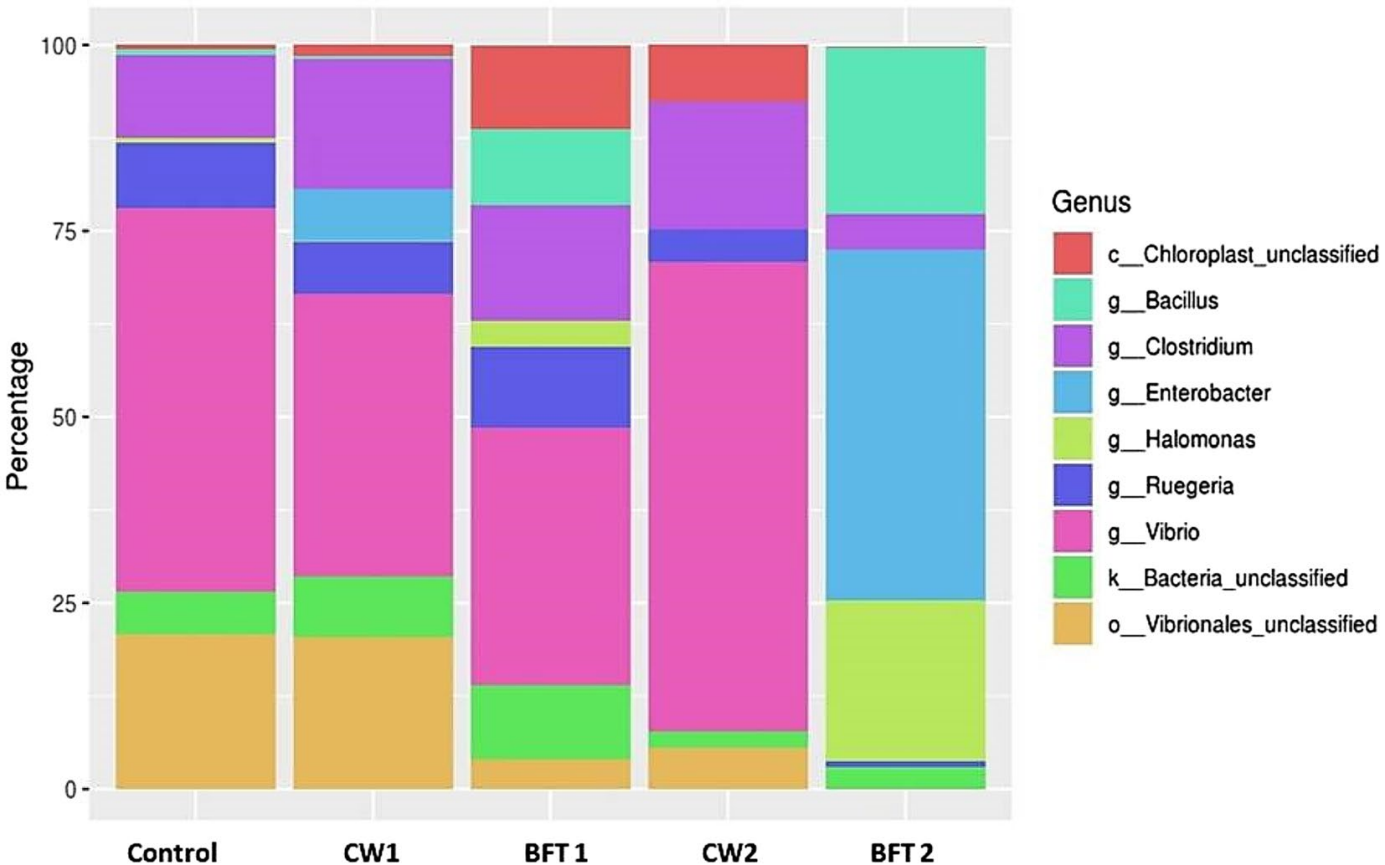
Dominant species abundance distribution.

**Figure 3:**
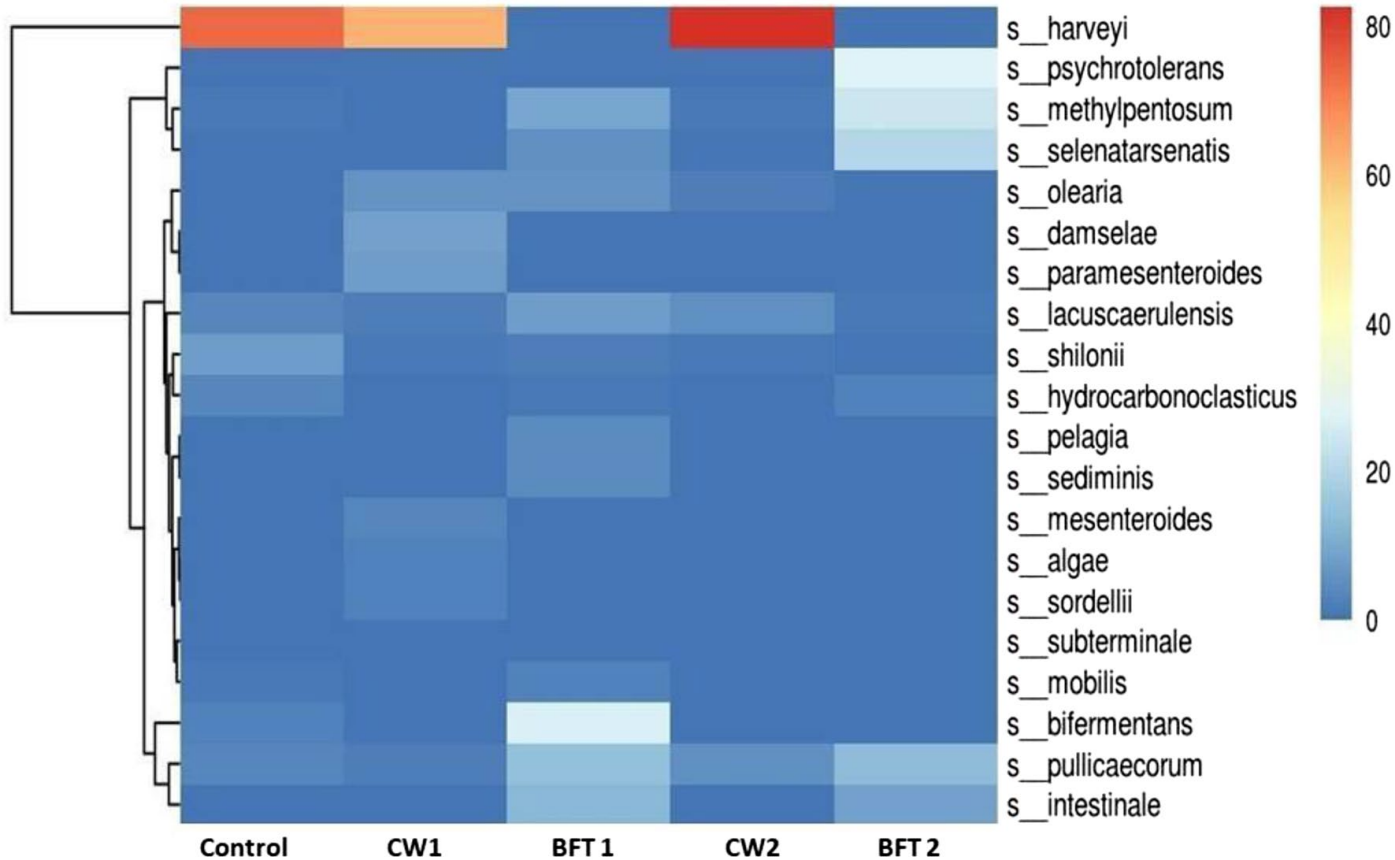
Heatmap representing distribution of top 20 species.

Microbial community is a nascent constituent of biofloc based aquaculture and the structure and function of microbial community highly influences the growth and survival rate. Culturable microbes can be identified with conventional methods but uncultured microbial communities are also taken into account and evaluated through metagenomics analysis of V3–V4 hypervariable region of 16 s rRNA by using high throughput sequencing approaches. The results of the high throughput sequencing analysis of gut samples taken from different treatments showed varied microbial population among clear water culture, biofloc culture and control. Many of the researchers have explored the microbial communities in biofloc system which influences the functional quality of the same. But in recent studies, it was inferred that gut of biofloc reared animals was also having important role in the host physiological activities by producing variety of enzymes^31^. So the microbial variation in gut samples of different treatments was analyzed and the major phyla of different treatments are depicted in Table 2. *Proteobacteria* and *Firmicutes* are the predominant phyla observed in the treatments and they were higher in bio-augmented systems than the control. *Bacteroidetes* and *Planctomycetes* were the other dominant microphyla observed in the treatments. Figure 2 represents the dominant genus observed in the treatment and control. *Vibrio* dominance was high in control and clear water system compared to that of BFT system. This phenomenon of heterotrophic bacterial community inhibiting the *vibrio* population was successfully demonstrated through the 16 s high throughput sequencing approach in our previous study^25^. The genus *Enterobacter* was also observed to be high in biofloc system especially BFT 2. The next observed genera were *Bacillus*, which was greatly aggregated in biofloc treatments than clear water system of strain supplementation. *Bacillus* genera in lesser amount were observed in control culture. *Ruegeria,* a genus of the *Rhodobacteraceae* family is a marine *Agrobacterium* which was observed to be higher in BFT2 group followed by BFT 3. *Halomonas* genus was observed majorly in BFT 2 and CW2 than others (Fig. 2). Top 20 major bacillus genera were observed in different treatments and control (Fig. 3).

**Table 2:** Major Phyla abundance among the samples.

Phylum	Control	CW1	BFT1	CW2	BFT2
Proteobacteria	12,755	23,153	20,981	18,473	31,152
Firmicutes	8104	31,329	11,588	17,798	14,489
Bacteroidetes	2113	4651	2703	293	2779
Planctomycetes	499	1659	4329	193	3140
Cyanobacteria	113	3871	524	600	3274
Bacteria_unclassified	729	2881	1542	663	800
Actinobacteria	343	2188	342	1147	175
WS6	42	358	235	9	1239
Chloroflexi	136	542	650	30	296
Verrucomicrobia	45	509	749	21	282

In the present study, 16 s High throughput sequencing approach was followed and the analysis of gut samples revealed considerable variation in control, clear water with probiotic supplement and biofloc with probiotic supplement. Rarefaction curves for species richness in all five samples plateau at the maximum depth, indicating an adequate sampling was conducted (Supplementary Fig. S1). The bacterial diversity in each treatment, alpha diversity (ACE, Chao and Shannon) was analyzed. The richness index ACE varied from 375.03 to 604.40 and Chao1 varied from 363 to 605.92. The diversity index Shannon varied from 2.81 to 3.92. All the indices showed significantly ascending trend in BFT1 group (Supplementary Table S1). Microbial colonization and its function are usually attributed to the host nutrient and environmental status^25,30,31^. Among all the treatments, *Proteobacteria* was the major phylum observed followed by *Firmicutes* and *Bacteroidetes*; these populations are highly colonized in biofloc treatments. Many marine proteobacterial populations are found to produce valuable natural bioactive compounds like astaxanthin, haliangicin, ecotin, bryostatins etc. and these compounds benefit nutritionally and physiologically to the host animal^32^. Also, the colonization depends on the type of carbon sources supplied. Wei et al.^33^ observed that biofloc produced by using glucose, starch and glycerol was majorly dominated by *Proteobacteria* and *Bacteroidetes,* an observation corroborated by the present study. Martínez-Córdova et al.^34^ observed that *Planctomycetes*, *Proteobacteria* and *Bacteroidetes* are the dominant bacterial phyla observed in biofloc produced with amaranth meal whereas biofloc produced with wheat harbored *Bacteroidetes* as the dominant phyla.

### Gene expression

**Figure 4:**
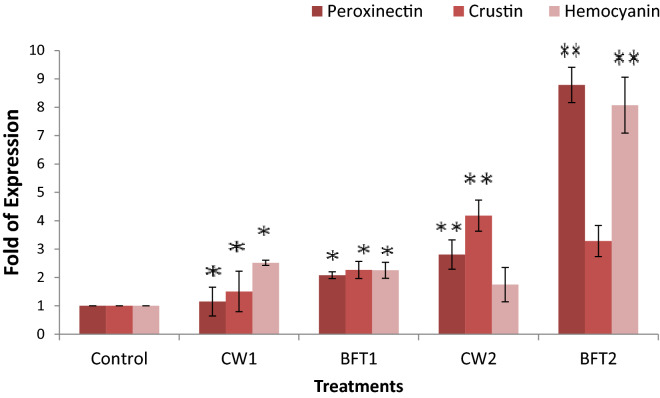
Relative expression of peroxinectin, crustin and hemocyanin of *P. indicus* cultured in different treatments and error bar showing standard deviation of three replicates. Significance between different groups (*P* < 0.05) marked with asterisk**.**

**Figure 5:**
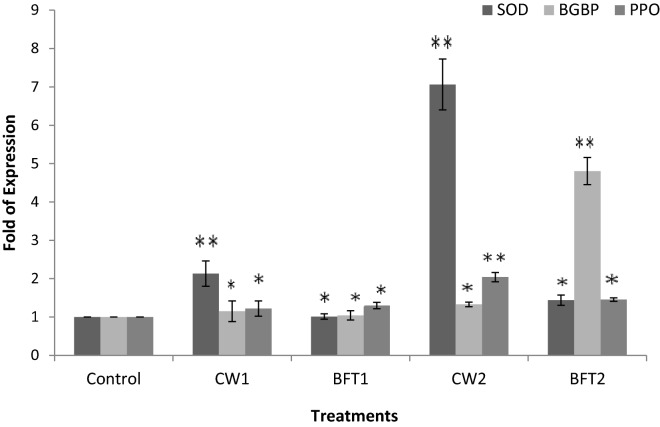
Relative expression of SOD, β-glucan binding protein and prophenol oxidase of *P. indicus* cultured in different treatments and error bar showing standard deviation of three replicates. Significance between different groups (*P* < 0.05) marked with asterisk.

Effect of the bio-augmentation of two strains on the immune status revealed an improved health status of the animals reared in these systems compared to that of the control. The effect was more significant in biofloc system when compared to clear water system. Also, the digestive system of the animal was highly influenced in the biofloc system revealing a positive effect as inferred by using expression of digestive enzyme related genes. A significant alteration of immune related genes was observed with supplementation of two strains in clear water and biofloc system. Peroxinectin and hemocyanin mRNA transcript upregulation was significantly higher (*P* < 0.01) in BFT2 and an eight-fold increase was observed compared to that in control. The expression of antimicrobial peptide crustin gene was observed to be upregulated in CW2 (four fold) than BFT 2 (three fold) (Fig. 4). The expression of SOD gene was observed to be high in CW2 and CW1 than biofloc treatments and control. The β-glucan binding protein was significantly upregulated (P < 0.01) in BFT2 and it was five-fold higher than in control. In other treatments a marginally higher expression was observed compared to that in control. The expression of prophenol oxidase (proPO) was also upregulated with these two strains added treatments and it was better in CW2 (Fig. 5).

One of the most advantageous benefits of the biofloc system is improving the immunity. In the present study, the expression of selected immune related genes was studied based on the effect of two different strains in conventional and biofloc systems. It has been reported that the transcriptional response of immune genes was highly induced by bioflocs^25,35,36^ and the results of our study revealed that the biofloc system combined with live microbial supplementation efficiently improved the immune status. Significant improvement of prophenoloxidase, β-glucan binding protein and peroxinectin expression was observed in both the strain supplied systems than the control. Prophenol oxidase system is a notable shrimp innate immune system associated with melanization. This can be activated by many factors including β-glucan binding protein and peroxinectin. Peroxinectin cell adhesion molecule plays a significant role in immunity^37,38^. The expression of these two confers the efficacy of live microbial supplementation in biofloc system. The other observed parameters like antimicrobial peptide, crustin and antioxidant enzyme SOD were also found to be significantly expressed in the system where probiotic strains were added. It is generally reported that the antimicrobial status and antimicrobial activity are important factors in building immunity. The higher expression of SOD, crustin and hemocyanin results in a higher antioxidant enzyme activity and antimicrobial status. Aguilera-Rivera et al.^39^ reported that biofloc rearing of *P. vannamei* shrimp considerably increased the SOD and hemocyanin transcripts and provided protective response against *Vibrio harveyi*. Cardona et al.^40^ reported that biofloc rearing was more beneficial in improving antioxidant and antimicrobial defense in *Litopenaeus stylirostris.*

**Figure 6:**
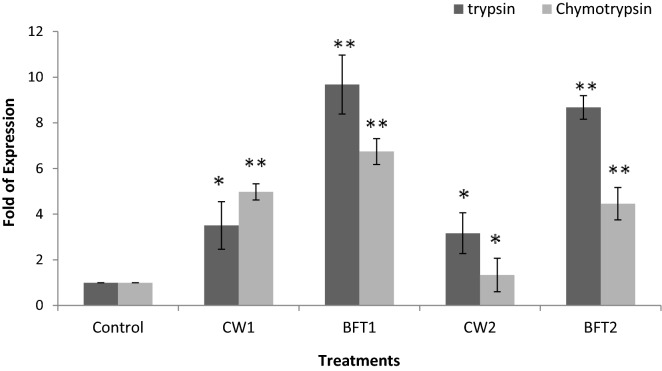
Expression level of trypsin and chymotrypsin of *P. indicus* cultured in different treatments and error bar showing standard deviation of three replicates. Significance between different groups (*P* < 0.05) marked with asterisk**.**

**Figure 7:**
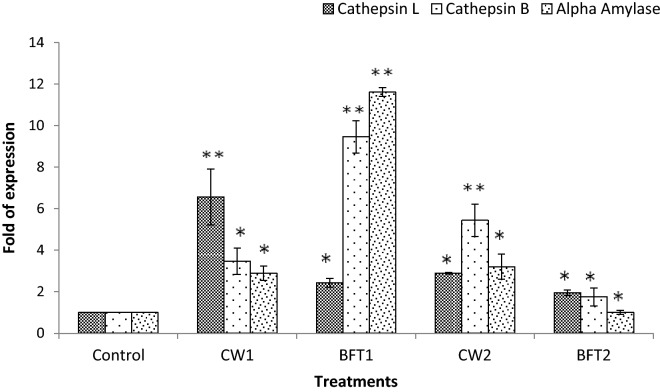
Expression level of cathepsin L, cathepsin B and alpha amylase of *P. indicus* reared in different treatments and error bar showing standard deviation of three replicates. Significance between different groups (*P* < 0.05) marked with asterisk.

The efficacy of different strains supplemented on the digestive enzyme activities of *P. indicus* was analyzed using the expression of mRNA transcripts of different enzymes such as trypsin, chymotrypsin, cathepsin L, cathepsin B and alpha amylase. The results in general, suggested that mRNA transcript of the selected enzyme genes were significantly (*P* < 0.05) upregulated in treatments supplemented with strains and among these, probiotic strains with biofloc system had higher levels of these enzymes as evident from the upregulation of the concerned genes. A three-fold upregulation of trypsin was observed in BFT1 and BFT2 compared to CW1 and CW2. The chymotrypsin level was also substantially upregulated. In case of cathepsin L, it was expressed at a higher level in CW1 and CW2 than BFT1 and BFT2. Cathepsin B was upregulated in BFT 1 compared to CW1, but the same was downregulated in BFT 2 compared to CW2. The level of alpha amylase expression was very high in biofloc reared animals than the clearwater reared shrimps (Figs. 6 and 7).

As stated above, biofloc system continuously supplies viable nutrients throughout the culture period and these paves the way in stimulating the digestive status of the host animal. In the present study, the effect of two different bacterial strains on the digestive enzyme status of the shrimp was studied by qPCR. The level of selected enzymes trypsin, chymotrypsin, alpha amylase, cathepsin L and cathepsin B were upregulated significantly (*P* < 0.05) in biofloc system. Cardona et al.^40^ reported that digestive enzyme activities of *L. stylirostris* was significantly influenced in transcript and enzymatic level with biofloc system compared to clear water system and without biofloc, which is consistent with our findings. Our previous study revealed the benefits of biofloc based system over the conventional system and the manner in which the microbial community plays a vital role in inducing nutritional immunomodulation^2,31^. Xu and Pan^41^ opined that biofloc system stimulates the activity of digestive enzymes such as amylase and protease in *P. vannamei* which resulted in improved growth.

### Proteomic analysis

**Figure 8:**
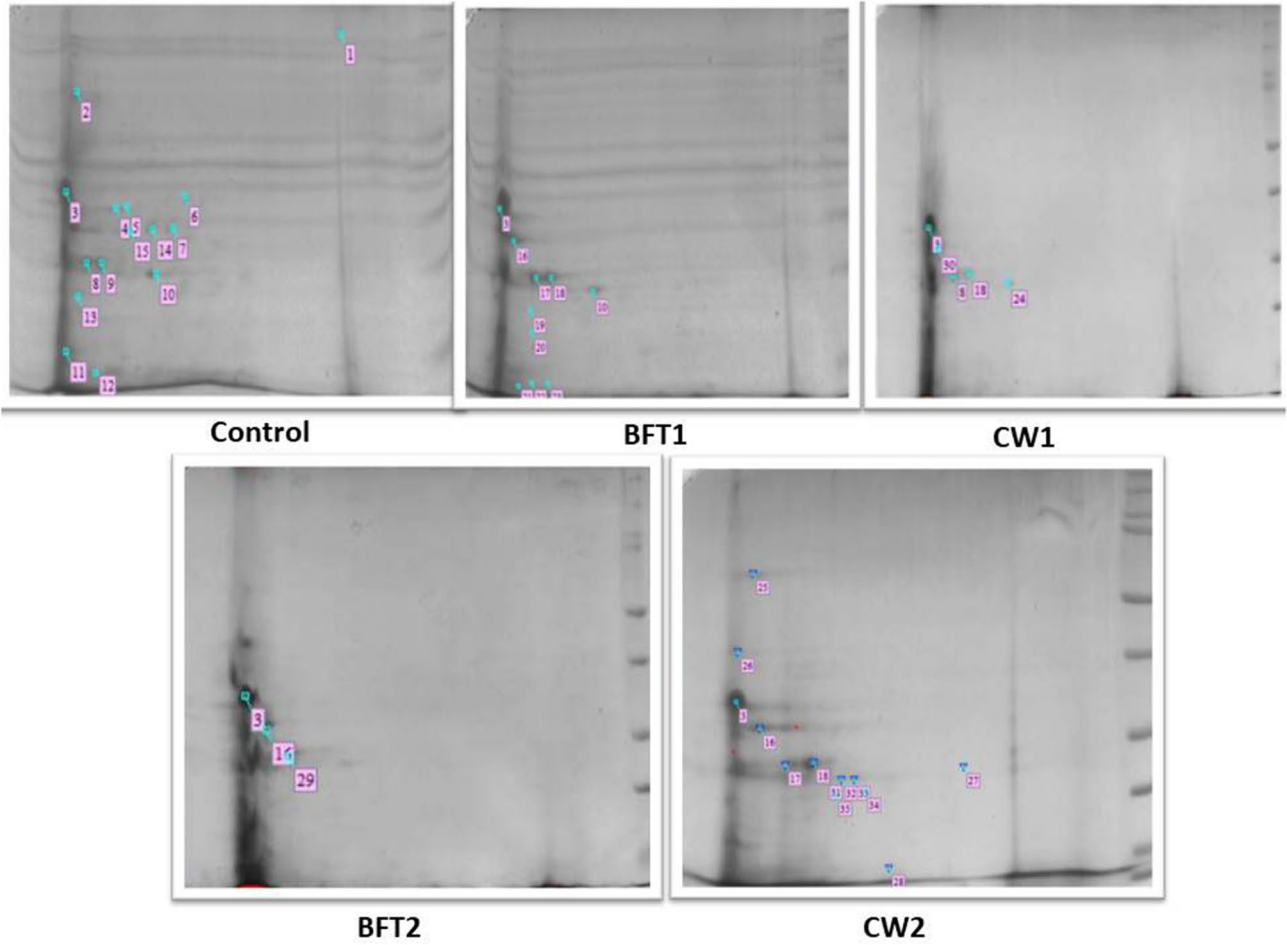
2D gel electrophoresis of hepatopancreas samples taken from different treatments (uncropped whole blots of all replicates are provided in Supplementary Fig. S2).

Proteomic analysis of hepatopancreas of different samples was carried out by using 2D gel electrophoresis and MALDI-TOF analysis. The results revealed that different proteins were expressed in different treatments that the biofloc reared animals were exhibiting less proteins spots than clear water group animals and control. The proteins mostly related to growth and stresses were observed in clear water and control group animals. Eukaryotic initiation factor 4E binding protein was observed to be expressed in all the groups and it was high in biofloc treated animals followed by probiotic alone group and control. It was reported that proper dietary protein supplementation would directly influence the protein synthesis and deposition. The protein synthesis included initiation, elongation and termination^42^. Hence, the high expression of Eukaryotic initiation factor 4E binding protein in BFT groups indicated the optimum nutrient availability through optimum C:N ratio that confers the growth. Kelch motif family protein was observed to be upregulated in BFT 1 and BFT 2 followed by CW2. It was not observed in control and CW1 (Fig. 8 and Table 3, Supplementary Table S2; Supplementary Fig. S2). These proteins are identified as propellers of cell function and having diverse functions in the cells^43^. They also play an important role in maintaining the cell integrity during stress conditions and also in signal transduction protein during WSSV infection^44,45^.

**Table 3:** Class analysis of differently expressed proteins from different samples.

Match ID	Spot ID	Max	Match Count	Control	BFT1	CW1	BFT2	CW2	Anova F-value
0	10	5.59246	2	3.25658	5.59246				7.61E−05
1	8	13.6974	2	5.25012		13.6974			3.12E−06
2	9	3.43556	1	3.43556					1.38E−15
3	17	9.90383	2		9.90383			5.52283	0.00212005
4	1	2.41413	1	2.41413					0.0786346
5	2	4.777	1	4.777					5.16E−04
6	25	6.35778	1					6.35778	3.64E−05
7	3	34.6689	5	18.4798	33.4806	32.7215	34.6689	30.3157	0.635544
8	6	1.38421	1	1.38421					2.56E−07
9	4	2.00904	1	2.00904					3.00E−05
10	5	1.24315	1	1.24315					0.00818628
11	7	1.94239	1	1.94239					1.69E−14
12	14	1.94804	1	1.94804					2.33E−07
13	15	2.22911	1	2.22911					8.74E−08
14	13	3.54775	1	3.54775					6.40E−06
15	16	18.1157	3		18.1157		13.1128	8.8146	0.0221414
16	18	6.85196	3		4.85939	6.85196		6.37793	0.0190542
17	11	14.8721	1	14.8721					3.48E−06
18	20	1.32787	1		1.32787				0.00127915
19	19	1.11502	1		1.11502				0.0625564
20	21	3.81292	1		3.81292				6.99E−04
21	22	3.37753	1		3.37753				0.0260651
22	23	1.36928	1		1.36928				0.00425162
23	26	9.4883	1					9.4883	4.04E−07
24	12	5.98229	1	5.98229					1.09E−05
25	29	5.96899	1				5.96899		0.0347055
26	28	6.25922	1					6.25922	0.117992
27	30	22.0581	1			22.0581			2.93E−11
28	27	1.70357	1					1.70357	4.43E−05
29	24	6.11321	1			6.11321			4.02E−05
30	31	0.277464	1					0.277464	0.00641368
31	32	0.285727	1					0.285727	6.62E−04
32	33	2.50462	1					2.50462	0.0784023
33	34	0.0524704	1					0.0524704	1.77E−04

It is important that the expression of hypoxia inducible factor 1a was exclusively observed in BFT1 group animals. These transcription factors are important to regulate many genes in response to hypoxia^46^ which increases the susceptibility of shrimp to WSSV infection^47^. Structural maintenance of chromosome protein is only expressed in BFT 1 animals which are responsible for maintenance of chromosome transmission during replication and segregation. Serine proteinase was expressed only in BFT2 group animals which is a major enzyme also responsible for immunity as observed in *P. monodon* during *V. harveyi* infection^48^. At the same time, Alpha-amylase OS are only observed in BFT1 animals. Vacuolar protein sorting-associated protein 29 OS was reported to have a relationship with the immune response of insects^49^ which is observed only in CW2 group animals. Hemocyanin is a multifunctional oxygen-carrying protein immune defense factor reported to have antibacterial and antiviral defense properties^50,51^. This was considerably upregulated in CW2 group animals than control, whereas the animals in other groups do not show the expression of the same. Similarly, Serine proteinase inhibitor 6 was observed in CW2 group animals only and this protein plays a role in controlling many biological processes as well as host defense mechanism such as pathogen digestion^52^.

It is therefore concluded that biofloc technology is an efficient technology for aquaculture and additional supplementation of probiotic strains to the biofloc would enhance the efficiency. This has been inferred from the analysis of differentially expressed proteins as well as the expression of immune and digestive enzyme related genes, in addition to a well accounted advantage in terms of growth, immunity and survival.

## Materials and methods

### Selection of strain

The bacterial strain-*Marinilactibacillus piezotolerans* strain AP BFT8 (MK934556) and *Novosphingobium* sp. strains AP BFT4 (MK934552) were selected for this study. The strain was isolated from *P. indicus* previously reared on biofloc based system with different carbon sources. These strains were selected from a total of 94 isolated colonies based on its extracellular enzymatic (amylase, lipase, protease) profile and antibiofilm activity against pathogenic *Vibrio* sp.^31^.

### Experiment

A 45 days experiment was conducted at the Muttukadu Experimental Station of ICAR-CIBA. The candidate species *P. indicus* (average weight 1 g) was stocked in 500 L tanks at the rate of 100 no/per tank. The effect of these two strains were evaluated in a biofloc system and compared with shrimps reared in a clear water system. The experimental combinations were as follows: CW1 (*Novosphingobium* sp. in clear water), BFT 1 (*Novosphingobium* sp. + biofloc), CW2 (*M. piezotolerans* in clear water), BFT 2 (*M. piezotolerans* + biofloc) and a treatment devoid of these strains and biofloc were taken as control (C). The experiment was conducted in triplicate.

Prior to stocking, tanks were prepared for the floc development. Initially the tanks were filled with disinfected (Bleach@60 ppm) sea water, for developing autotrophic bloom the fertilizer such as (Urea (3 g/m^3^), Triple super phosphate (0.5 g/m^3^), and dolomite (5 g/m^3^) were added in an alternate days for 3 days, then the aerobically fermented biofloc inoculum were added to appropriate tanks regularly till the floc level reaches upto 2–5 ml/L to convert the autotrophs into heterotrophic medium. Biofloc inoculum was prepared by fermenting a mixture of carbon sources and bacillus microbial consortium (CIBA-floc) and allowed to proliferate; the filtrate of this was added to the biofloc treatment tanks at the rate of 1 L/m^3^. Thereafter, the animals were stocked and fed with commercial nursery shrimp feed (Inve Aquaculture, Thailand) at 10% of the body weight in each tank. Biofloc was added to the respective tanks on alternate days. The water was added at 10% per week to compensate the evaporation loss in the experimental tanks whereas 50–60% water was exchanged in control and clear water system tanks per week. The probiotic strains were grown on liquid broth medium (peptone-0.5%, yeast extract-0.1%, KH_2_PO_4_-0.05%, sodium nitrate-0.1%, sodium chloride-2% and dextrose-0.1%). Thereafter, the respective probiotic strains were inoculated and kept in a shaker at 150 rpm at 32 °C. After reaching the OD at 600 nm, the cultures were inoculated to the respective tanks at the rate of 20 mL per tank (0.1%). The probiotic strains were added once in 2 days. Sampling of shrimps was done once in 15 days to monitor and record growth and survival.

### Analysis of water quality, growth and survival

Physico-chemical parameters such as salinity, pH, DO, ammonia nitrogen, nitrite, and phosphate were measured periodically by following standard methods described in APHA (1995). During sampling, the length, weight and survival were recorded once a fortnight, and the survival was calculated as the ratio of the number of shrimps survived to the number of shrimps stocked × 100, and weight gain was computed as the difference between the final and initial average weights.

### Gut microbial analysis

At the end of the experimental period, shrimp gut samples were collected by aseptically dissecting 5 animals from each experimental group. Total bacterial DNA was extracted from the gut samples using a QIAamp DNA stool mini kit according to the manufacturer's protocol. An amount of 25 ng of DNA was used to amplify 16S rRNA hyper variable region V3–V4. The reaction included KAPA HiFi HotStart Ready Mix and 100 nm final concentrations of modified 341F (3′ CCTACGGGNGGCWGCAG-5′) and 785R (3′-GACTACHVGGGTATCTAATCC-5′) primers. The PCR involved an initial denaturation of 95 °C for 5 min followed by 25 cycles of 95 °C for 30 s, 55 °C for 45 s and 72 °C for 30 s and a final extension at 72 °C for 7 min. The amplicons were purified using Ampure beads. Additional 8 cycles of PCR were performed using Illumina barcoded adapters to prepare the sequencing libraries. The sequence data quality was checked using FastQC and MultiQC software.

After sequencing, the degenerate primers were removed by trimming (20bp) from 5′ end and the adapter sequences were removed using Trimgalore (Babraham, 2004) and further imported to mothur software and aligned to form contigs. After screening for errors, 300 bp and 532 bp sequence lengths were retained. The non-specific regions were aligned with 16s rRNA database. Thereafter, the gaps and overhang in the end region of the contigs were removed and the possible chimera sequences were removed by using UCHIME algorithm^53^. Further, it was classified into taxonomical outline and clustered into operational taxonomic unit using GREENGENES v.13.8-99 database^54^.

### Immune response

Expressions of immune related genes were analyzed in hepatopancreas samples and the selected genes were prophenoloxidase, ß-glucan binding protein, hemocyanin, crustin, SOD and peroxinectin. The digestive enzyme related genes such as trypsin, chymotrypsin, cathepsin L, cathepsin B and alpha amylase were also analyzed**.** Total RNA from hepatopancreas was isolated using TRIzol reagent (RNAiso plus, Takara) and converted into cDNA using Prime Script 1st Strand cDNA Synthesis Kit (BioRad, USA). Thereafter, the cDNA was serially diluted for further relative quantification of target immune genes. The primers used are listed in Table 4. Total 20 μL of reaction constituted [10 μL of 2 × SYBR Green qPCR master mix (Bio-Rad, USA), 1 μL each of forward and reverse primers (10 pmol), 1 μL of template DNA (30–60 ng) and 7 μL of PCR-grade water]. PCR was performed in Real-time PCR (Applied Biosystem's Real-Time PCR system StepOne Plus). The following conditions were maintained; holding stage of 10 min at 95 °C (initial denaturation), followed by 45 cycles of 15 s at 95 °C (denaturation), and 1 min at 60 °C (annealing and extension). Expression of ß-actin gene was used as an internal control and the data were analyzed using cycle threshold (CT) method^55^.

**Table 4:** List of primers used for real-time PCR (qPCR).

Gene	Primer sequence (5′–3′)	Accession no./Reference
Trypsin	F-TCCTCTCCAAGATCATCCAA R-GGCACAGATCATGGAGTC	Stephens et al., 2012
Chymotrypsin	F-GGCTCTCTTCATCGACG R-CGTGAGTGAAGAAGTCGG	Stephens et al., 2012
Cathepsin L	F-CTCAGGACGGTAAGTGTCG R-TTCTTGACCAGCCAGTAGG	Stephens et al., 2012
Cathepsin B	F-GGATGTAACGGAGGCTTC R-CTGTATGCTTTGCCTCCA	Stephens et al., 2012
α-Amylase	F-GGTAAACACTGACTCACGCC R-TTCACGTCTCCCTGGTACAC	AH013375
ß-actin	F-CAACCGCGAGAAGATGACAC R-TCGGTCAGGATCTTCATCAGG	GU732815
Crustin	F-ACGAGGCAACCATGAAGG R-AACCACCACCAACACCTAC	AF430076
ProPO	F-TTCCAGCTCTTCTTCATGCT R-TCGGGGTACTTGGCGTCCTG	AY723296.1
SOD	F-GCTGAATTGGGTGAGGAACG R-CCTCCGCTTCAACCAACTTC	AY486424
Hemocyanin	F-GCTTTTCGACGTCCTCATCC R-CTTGAATTTGCCAGGCGTCT	X82502
Peroxinectin	F-GAGTCTGAACATCCATCGCG R-TATGCCACCCACGAAGAAGT	KC708021
ß-glucan binding protein	F-TTATACCCGAGACTCCACGC R-ACGTCCGTATCTGAAAGCGA	AY723297.1

### Proteomic analysis

Hepatopancreas sample were taken from shrimps belonging to varied experimental groups and proteins were extracted by using lysis buffer (135 mM NaCl, 2.7 mM KCl, 1.5 mM KH2PO4, and 8 mM K2HPO4, 0.1% protease inhibitor cocktail mixture pH 7.2) in liquid nitrogen. For two-dimensional gel electrophoresis of processed proteins (150 µg), 13 cm IPG strips of pH 4–7 (GE Healthcare, Uppsala, Sweden) were employed in the first dimension^56,57^. Passive Rehydration process was performed and proteins were focused for a total of 50,000 Vhs at a constant temperature (20 °C) under linear voltage ramp after at 30 V in a PGPhor III (GE Healthcare, Uppsala, Sweden) apparatus with following IEF conditions, 100 V gradient for 1 h, 300 V gradient for 2 h, 1000 V gradient for 1 h, 5000 V gradient for 5 h and 5000 V step on hold for 7 h. Following IEF, each IPG strip was placed in the equilibration buffer containing 2% DTT first followed by incubation in another buffer in which the DTT was replaced by 2.5% iodoacetamide. The second dimension PAGE (12.5%) was carried out in an SE600 (GE Healthcare, Uppsala, Sweden) at 1 W/gel for 1 h and 13 W/gel for 3 h. Further, different spots from each sample were picked and analyzed by using MALDI-TOF and similarity search was made using Mascot search engine.

### Statistical analysis

The data obtained from the experiment were analyzed by SPSS (Version-17). The comparison between all the treatment groups and between the treatments (P < 0.05) was made using one way ANOVA and Duncan's multiple range test (DMRT), respectively. The probability level was kept at 5% for the statistical analysis.

### Ethics declarations

Ethics approval: The research undertaken complies with the current animal welfare laws in India. The study was undertaken with the approval of the statutory authorities of the Central Institute of Brackishwater Aquaculture, Chennai, India. The experimental animal *Penaeus vannamei* is not an endangered shrimp; the provisions of the Govt. of India's Wildlife Protection Act of 1972 are not applicable for experiments on this shrimp. Shrimp intestinal samples were collected by dissecting out the shrimp guts followed by homogenization in RNA/DNA shield (ZymoResearch, Irvine, CA, USA).

## Supplementary Information

Supplementary Information.
