# Supplementary material for: Bioaugmentation of biofloc system with enzymatic bacterial strains for high health and production performance of Penaeus indicus
Source: Sci Rep. 2021 Jul 1;11:13633. doi: 10.1038/s41598-021-93065-3 (PMC8249640; doi:10.1038/s41598-021-93065-3)
Supplement: Supplementary file 1 — Supplementary Information. [file 41598_2021_93065_MOESM1_ESM.docx]

**Bioaugmentation of biofloc system with enzymatic bacterial strains for high health and production performance of *Penaeus indicus***

A. Panigrahi*, P. Esakkiraj, R. R. Das, C. Saranya, T. N. Vinay, S. K. Otta, M. S. Shekhar

ICAR-Central Institute of Brackishwater Aquaculture, Chennai - 600 028, India

*Correspondence: Akshaya Panigrahi; apanigrahi2k@gmail.com

**Table S1.** Differentially expressed proteins identified by MALDI-TOF


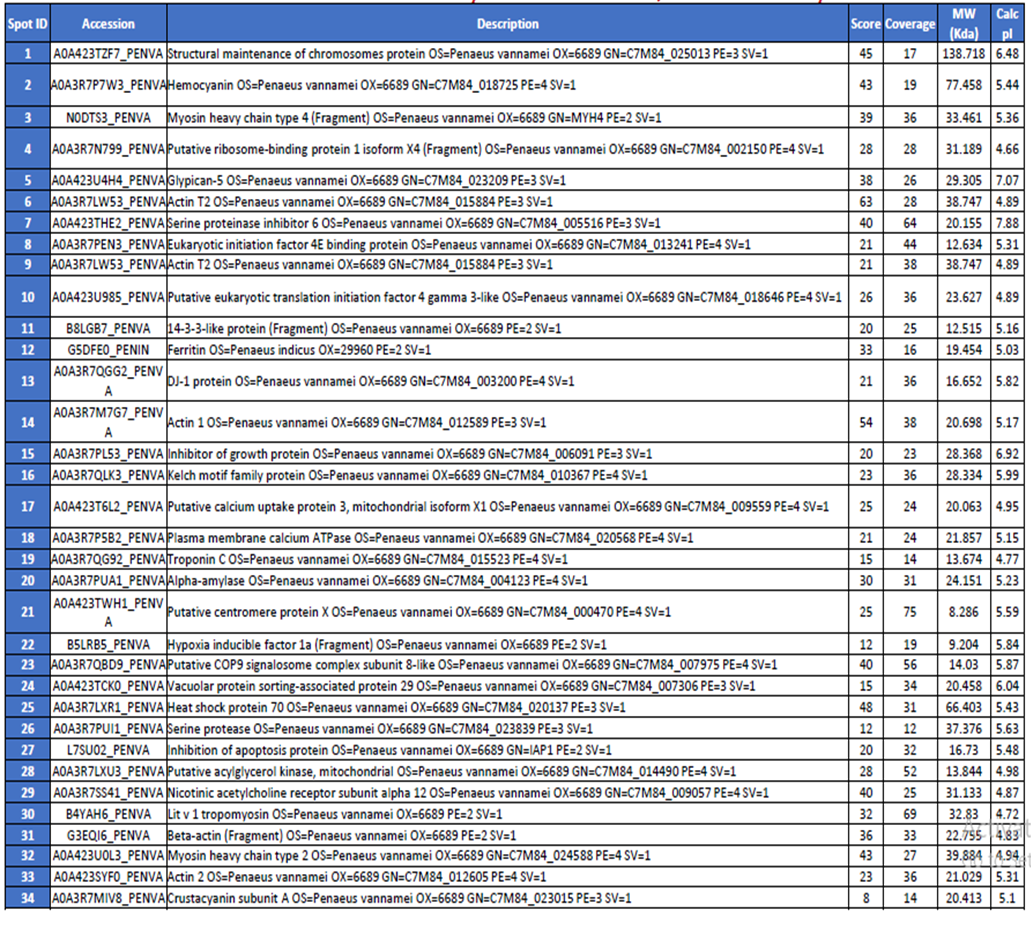


**Table S2.** Summary of alpha-diversity results for microbiota samples.

| Index | Groups | | | | |
| --- | --- | --- | --- | --- | --- |
|  | Control | CW1 | BFT1 | CW2 | BFT2 |
| ACE | 375.03 | 465.11 | 604.14 | 604.41 | 423.23 |
| Chao1 | 363.00 | 455.49 | 605.92 | 564.43 | 411.98 |
| Shannon | 3.4 | 3.85 | 3.92 | 3.00 | 2.82 |


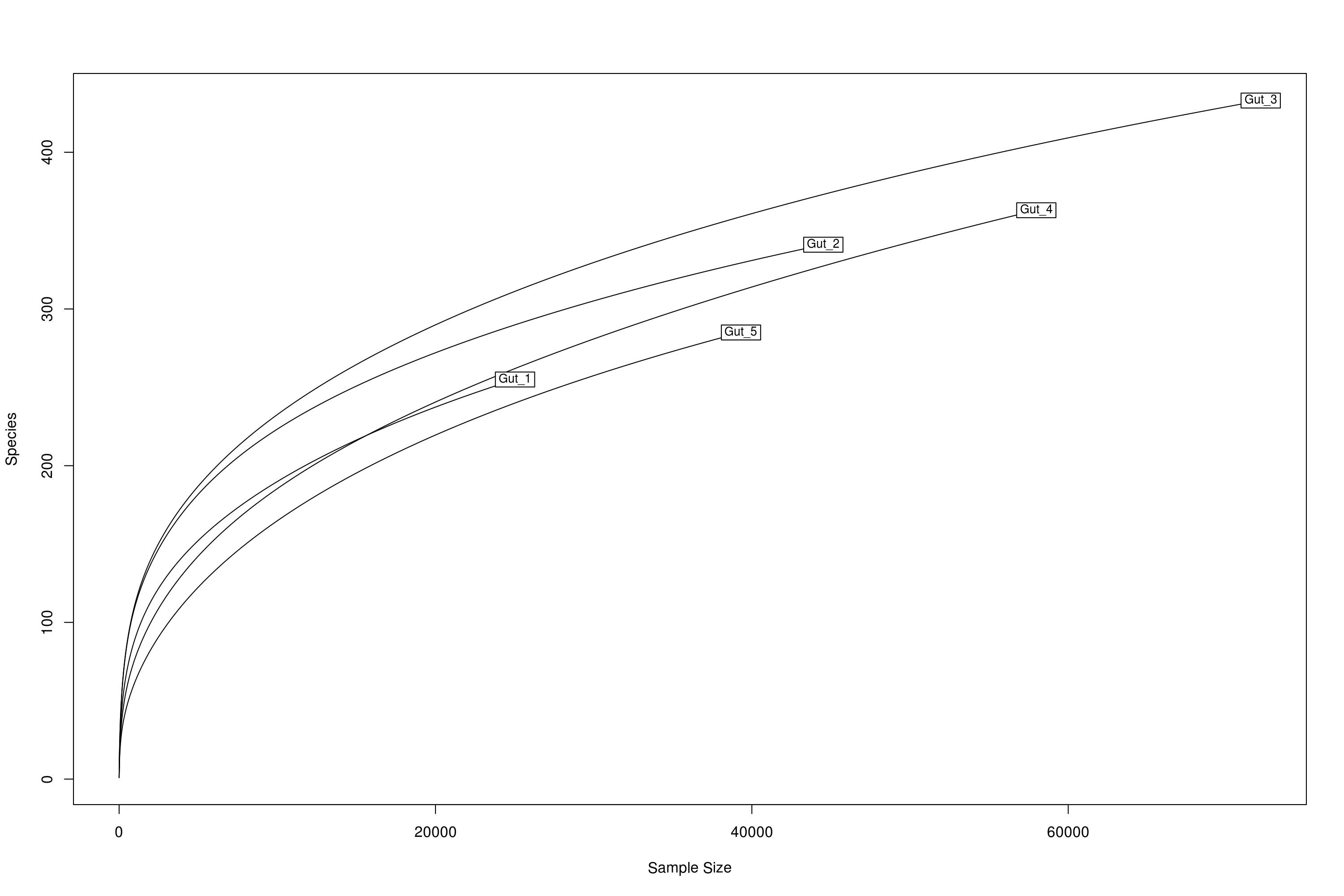


**Figure S1.** Rarefaction curves of five microbiome samples.

**
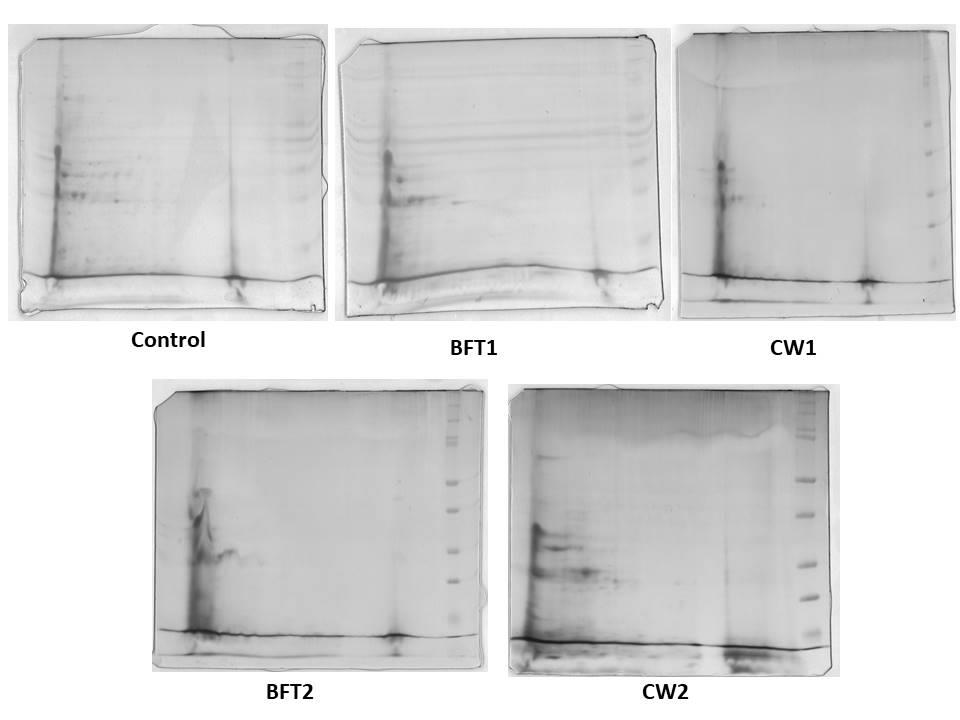
**

**Figure S2.** 2D gel electrophoresis of hepatopancreas samples taken from different treatments in triplicate**.**
